# Is it Necessary to Perform Sentinel Lymph Node Biopsy in Thin Melanoma? A Retrospective Single Center Analysis

**DOI:** 10.1007/s12253-019-00769-z

**Published:** 2019-12-02

**Authors:** A. Kocsis, L. Karsko, Zs. Kurgyis, Zs. Besenyi, L. Pavics, E. Dosa-Racz, E. Kis, E. Baltas, H. Ocsai, E. Varga, B. Bende, A. Varga, G. Mohos, I. Korom, J. Varga, L. Kemeny, I. B. Nemeth, J. Olah

**Affiliations:** 1 Department of Dermatology and Allergology, University of Szeged, Szeged, Hungary; 2 Department of Nuclear Medicine, University of Szeged, Szeged, Hungary; 3 Department of Oncology, Faculty of General Medicine, University of Szeged, Szeged, Hungary

**Keywords:** Melanoma, Sentinel lymph node biopsy, Mitotic rate, Regression, Stage

## Abstract

Sentinel lymph node biopsy (SLNB) is a standard procedure for regional lymph node staging and still has the most important prognostic value for the outcome of patients with thin melanoma. In addition to ulceration, SLNB had to be considered even for a single mitotic figure in thin (<1 mm) melanoma according to AJCC7th guideline, therefore, a retrospective review was conducted involving 403 pT1 melanoma patients. Among them, 152 patients suffered from pT1b ulcerated or mitotic rate ≥ 1/ mm^2^ melanomas according to the AJCC7th staging system. SLNB was performed in 78 cases, of which nine (11.5%) showed SLN positivity. From them, interestingly, we found a relatively high positive sentinel rate (6/78–8%) in the case of thin primary melanomas ˂0.8 mm. Moreover, the presence of regression increased the probability of sentinel positivity by 5.796 fold. After reassessing pT stage based on the new AJCC8^th^, 37 pT1b cases were reordered into pT1a category. There was no significant relation between other characteristics examined (age, gender, Breslow, Clark level, and mitosis index) and sentinel node positivity. Based on our data, we suggest that mitotic rate alone is not a sufficiently powerful predictor of SLN status in thin melanomas. If strict histopathological definition criteria are applied, regression might be an additional adverse feature that aids in identifying T1 patients most likely to be SLN-positive. After reassessing of pT1b cases according to AJCC8^th^ regression proved to be independent prognostic factor on sentinel lymph node positivity. Our results propose that sentinel lymph node biopsy might also be considered at patients with regressive thin (˂0.8 mm) melanomas.

## Introduction

Although accounting for less than 5% of skin cancers, malignant melanoma is responsible for 80% of skin cancer-related deaths, and its incidence is increasing worldwide [1]. Because of increased awareness and earlier diagnosis, approximately 80% of new cases are in situ or early invasive melanomas. The prognosis for these patients is relatively good, with ten-year survival rates of more than 90% [2–4].

The result of the Multicentre Selective Lymphadenectomy Trial (MSLT-I) has been published, confirming the previous practice of performing sentinel lymph node biopsy (SLNB) in the case of intermediate and thick melanomas [5]. However, some authors already argue with the result or interpretation [6], highlighting some questionable points of the trial and debating the real benefit of the SLNB to ten-year melanoma-specific survival. The predictive role of sentinel lymph node positivity in thin melanoma has been investigated and reported by several authors [7–11]. There is a consensus in the literature that the metastatic involvement of regional lymph nodes is still one of the most important prognostic factors of thick and thin cutaneous melanomas as well, however, the criteria and the indications of thin melanomas for SLNB are inconsistent and the results both contradictory and incoherent [12].

In 2009, the American Joint Committee on Cancer (AJCC) published the 7^th^ edition of the staging system for melanomas which relies on thickness, ulceration and mitotic rate in the dermis. According to AJCC7^th^ recommendation, SLNB should be considered in the case of melanomas ≤1 mm in thickness, with ulceration or with even a single mitotic fig. [4]. The local guidelines for thin melanomas were modified at our department in early 2011 and the AJCC7^th^ adopted, as SLNB was offered not only if ulceration was present but if mitotic rate ≥ 1/mm^2^ of the tumour area. According to the new AJCC8^th^ guidelines, dermal mitoses are not considered for pT1b, however, mitotic activity should be noted as independent prognostic factor [13].

In our retrospective study, we aimed to investigate the predictive value of mitotic rate and to analyse further clinicopathologic predictors of positive SLNB by examining the common feature of these metastasizing cases in order to prevent the majority of the T1b melanoma patients from undergoing SLNB. Cases were also reassessed according to the AJCC8^th^ guideline and additional statistical analyses were performed to re-evaluate the prognostic value of the well-known histological parameters considering AJCC8^th^.

## Patients and Methods

The Department of Dermatology and Allergology, University of Szeged, is a regional centre for the management of cutaneous malignancies, being responsible for melanoma care for the 1.5 million inhabitants of south-eastern Hungary. A retrospective review was conducted involving patients treated at our department with thin melanomas (˂1 mm) between January 2011 and December 2014 *(Ethical approval: MEL-RETRO-001; number: 3521, 40/2015)*. During these four years, 625 consequtive primary melanomas were diagnosed at our department. Four hundred and three pT1 melanoma patients entered our study; among these cases, 152 patients suffered from pT1b ulcerated or mitotic rate ≥ 1/mm^2^ melanomas according to the AJCC7^th^ staging system. We also planned to evaluate the common and characteristic features of primary melanomas among thin melanoma cases with sentinel node involvement. On the basis of the histopathological results of the primary tumours, a multidisciplinary tumour board approved a therapeutic plan and the choice of SLNB was discussed with patients in all cases. SLNB was offered to most eligible patients with pT1b melanomas as part of their surgical management in the absence of clinically evident nodal disease, or known distant metastases. SLNB was not advised if any sign of dissemination was detected in the case of high biological age, severe comorbidities or pregnancy. Some patients had declined surgery. Seventy-eight cases of SLNB were included in the study based on the criteria which are listed below.

## Histopathology

A standardized histopathological examination was performed on samples of primary melanoma, re-excision and sentinel lymph node dissection. Specimens were fixed in 4% buffered formaldehyde embedded in paraffin, and 4 μm sections were placed on silanized slides. In addition to routine haematoxylin eosin staining (Leica ST5020), further immunohistochemistry (Leica Bond Max Autostainer) involved Melan-A (DAKO; mouse clone A103), HMB45 (Biocare; mouse monoclonal), and occasionally S100 (DAKO, rabbit monoclonal) antibodies in a dilution of 1:300, 1:200, and 1:2000, respectively. At least three serial sections were prepared from sentinel node specimens.

## Surgical Procedure

Sentinel lymph node biopsy (SLNB) for the staging of melanoma was first described in detail in 1992. We perform combined radiotracer and blue dye mapping as usual. On the day before the surgery, dynamic lymphoscintigraphy is performed with human albumin colloid (Sentiscint; FJC Institute, Budapest, Hungary, Medi-Radiopharma Ltd., Érd, Hungary) labelled with 99mTc pertechnetate, with 80% of the particles sized 100 to 600 nm. As part of the double staining, 0.5 to 2.0 ml vital blue dye (Byk-Gulden, Konstanz, Germany) is injected 15–20 min before the incision. During the operation, a handheld gamma probe (C-Trak, AEA Technology, Darwin House, Birchwood Park, Warrington and Navigator GPS System; RMD Instruments Corp. Watertown, MA 02472 USA) is used to identify the hot spots. The nodes with significant activity or with vital blue stain are considered sentinel lymph nodes. The radiotracer activity of these nodes is measured ex vivo and compared to the bed from which they were removed. A radioactive lymph node is defined as positive and removed until the background counts are <10% of the hottest node removed. If the postoperative histology reveals metastasis in the lymph nodes, completion lymph node dissection is offered to the patient.

## Statistical Methods

The SLN positivity rate was the primary outcome. The clinicopathological features analysed were age, sex, melanoma type, location, ulceration, Breslow thickness, Clark level, mitotic rate, regression, and SLN status. The relationships between SLN positivity and each of the clinical and histopathological parameters were assessed with Chi-square test, Student’s t test and Fisher’s exact test. Potential risk factors for SLN metastasis were analysed by multivariate logistic regression models with the enter method, and odds ratios (OR) and 95% confidence intervals (CI) were calculated for categorical variables. The predictive value of the multivariate logistic regression model was measured with the Nagelkerke R Square method. The relationship between the re-evaluated pT stage and the SNL status was assessed by Mann Whitney U test. *P* values <0.05 were considered to be statistically significant and all *p* values were two-sided. All statistical analyses were performed with the IBM SPSS Statistics Version 23.0 program.

## Results

According to AJCC7^th^ 152 patients with pT1b melanoma entered our study. Among these 152 cases, 74 patients underwent only local wide excision with a 1 cm safety margin. In addition of local wide excision SLNB was also performed in 78 cases. Twelve patients were excluded for previous cutaneous or other malignancies; the remaining patients were not involved due to high biological age, severe comorbidities or pregnancy, or because they had simply declined the procedure. Lymphoscintigraphy successfully identified the draining lymphatic basin and sentinel node in all 78 patients. The majority of patients were sentinel node-negative (*n* = 69); in nine cases (11.5%) metastasis was detected in the regional lymph nodes. The location, Clark level and mitotic rate of the tumour, the presence of ulceration, and regression were examined and compared. Complete lymph node dissection (CLND) was performed in 7/9 positive SLN cases. Additional metastatic LNs were found in two cases.

### Site of the Primary Tumour and SLN

With regard to the location of the primary melanoma, the majority of these tumours were found on the lower extremities in women and on the trunk in men, as we expected (Table 1).

**Table 1:** Location of primary tumours among all patients and in the SLNB group

Location	Overall male (*n* = 75)	Overall female (*n* = 77)	SLNB male (*n* = 34)	SLNB female (*n* = 44)
Head and neck	5	5	3	1
Trunk ventral	10	12	4	7
Trunk dorsal	33	21	14	13
Upper extremity	19	16	8	6
Lower extremity	7	22	4	16
Acral	1	1	1	1

Among patients who underwent SLNB, the most frequent location of the primary tumours was the dorsal region of the trunk (27/78 overall, 14/34 in men and 13/44 in women); however, a high proportion of the tumours were located on the lower limbs in the women (16/44). In our series, the SLN positivity was independent of the location of the primary tumour (Fisher’s exact test *p* = 0.9312).

As regards the location of the sentinel nodes, 46 patients had axillary SLNB, in seven cases from both sides. Six of these 46 patients had metastatic lymph nodes. Three patients had sentinel nodes from both the axillary and inguinal regions; one of these patients had a positive sentinel node. In 22 cases, the nodes were removed from the inguinum (1 positive) and in the case of two patients from the popliteal region as well (1 positive). Four patients had sentinel nodes in the neck, and one patient had a sentinel node at an atypical site (over the scapula).

### Age and Gender

The overall male/female ratio was 1:1. Of the patients who underwent SLNB, 43.5% were male, and the median age was 48.5 years (range 20–77 yrs). The onset of melanoma diagnosis did not differ significantly in the node-negative (48.8 yrs) and node-positive (46.3 yrs) groups. However, the mean age with pT1b/nodal involvement was 58.2 and 31.5 years among men and women, respectively. Moreover, in the younger age group (<35 yrs) the SLN positivity rate was 22.2%, which is higher than the average in all cases. Demographic and histopathological characteristics of the SNLB group are shown in Table 2.

**Table 2:** Demographic and histological characteristics of the primary tumours with SLNB

Variable	All patients with SLNB	SLN-positive	SLN-negative	*p* value	OR
Total no.	78	9	69		
Age mean (years)	48.526	46.33	48.812	0.614^b^	
<35	18	4	14	0.136^a^	
36–49	21	0	21		
50–64	32	4	28		
>65	7	1	6		
Gender				0.492^a^	1.724 (0.426–6.985) ^c^
Male	34	5	29		
Female	44	4	40		
Histological type					
Superficial spreading	72				
Nodular	4				
Acral lentiginosus	2				
Location				0.9312 ^a^	
Head and neck	4	0	4		
Trunk ventral	11	2	9		
Trunk dorsal	27	4	23		
Upper extremities	14	1	13		
Lower extremities	20	2	18		
Acral	2	0	2		
Thickness (mean)	0.716	0.714	0.717	0.966^b^	
Ulceration				0.586 ^a^	0^c^
Present	8	0	8		
Absent	70	9	61		
Mitotic rate (mean)	2.25	1.89	2.29	0.494 ^b^	
Clark level				0.715 ^a^	1.5 (0.368–6.114) ^c^
II	28	4	24		
III	50	5	45		
Regression				0.044 ^a^	4 (0.916–17.459) ^c^
Present	29	6	23		
Absent	49	3	46		
Reclassified stage (AJCC8^th^)				0.566 ^d^	
pT1a	37	4	33		
pT1b	41	5^e^	36		

^a^Fisher’s exact test

^b^Student t-test

^c^OR (95% CI)

^d^Mann Whitney U

^e^Two patients had melanoma with Breslow 0.76 mm, less than 0.8 mm, but ordered into pT1b group

α = 0.05

*p* two-sided

Multivariate logistic regression modelling demonstrates the association between SLN positivity and age, gender, Breslow, Clark level, and regression. The presence of regression in the primary tumour increases the probability of sentinel positivity by 5.796-fold. There was a significant correlation noted between histological regression and sentinel lymph node positivity, however, no significant relation between the other characteristics examined (age, gender, Breslow, Clark level, mitosis index; Nagelkerke R square = 0.7). After reassessing the pT stage according to the AJCC8^th^ guideline, 37 patients were reclassified from pT1b into pT1a category. By repeating the statistical analyses there was no significant association between reclassified stage and SLN positivity indicating that regression may have independent prognostic value on the lymphatic spread of melanoma (Table 3).

**Table 3:** Multivariate logistic regression model of the clinicopathologic parameters

Multivariate logistic regression model	SLN positivity	
Variable	OR (95% CI)	p
Age	.958 (.916; 1.002)	0.059
Gender	.651 (.152; 2.782)	0.562
Breslow	1.626 (.036; 72.656)	0.802
Clark	.560 (.112; 2.807)	0.480
Mitosis index	.723 (.348; 1.502)	0.385
Regression	5.796 (1.046; 32.123)	0.044 *

*OR* odds ratio

*CI* confidence interval

*p < 0.05 significant

method = enter

## Discussion

SNB has become standard procedure for the staging of the regional nodal basin in patients diagnosed with thin melanoma and remains one of the most important predictive factors of the outcome for these patients [7–11]. However, Morton et al. reported that local complications occur in approximately 10% of SNBs, a percentage which is not higher than other elective, clean surgeries [14]. Several previous authors have attempted to identify predictive risk factors for nodal metastases in thin melanomas, including Breslow thickness, ulceration, regression, Clark level, age, and tumour-infiltrating lymphocytes to prevent overtreatment of these patients. However, no widely accepted consensus exists as to which patients are at risk for nodal metastases.

In our study, we aimed to assess how efficiently melanoma staging systems can predict the occurrence of nodal metastases in thin melanoma and whether there are any other additional criteria to improve this rate.

### Age and Gender

Younger patient age is associated with a higher nodal metastasis rate among melanoma patients in general [3, 11, 15–18]; however, the available studies in thin melanoma are inconsistent on this factor, and there is no widely accepted specific age cut-off value under which SLNB should be performed. Kretschmer et al. reported that young patients (<40 years) in a series of 0.75–1.00 mm thin melanoma patients had a significantly higher SLN positivity rate than older age groups [19]. Sondak et al. have also reported that relatively young age (besides MR and Breslow depth) is associated with positive SLNs in melanoma patients [16]. In our study, we did not apply a particular cut-off age for SLNB (range 20–77 years). We placed emphasis on the characteristics of the tumour rather than on comorbidities or biological age. Corresponding to findings by Balch et al., male patients were slightly older than female patients (49.5 vs. 47.7 yrs.) [20]. However, our study did not identify any significant difference with regard to age among the SLN-positive and -negative groups. On the other hand, a marked difference was observed between male and female patients with metastatic SLNs. The mean age of SLN-positive men was 58.2 years versus 31.5 years among women. This might be the result of the small sample size of patients involved, and further investigation may be required.

### Breslow Thickness

The thickness of melanoma is generally considered the most useful prognostic factor in patients with thin melanoma. In a study of 121 thin melanoma cases, Hinz et al. [8] found that all SLN-positive patients belonged to the subgroup of tumour thickness range 0.9–0.99 mm. Han et al. [21] reported that a Breslow thickness of ≥0.76 mm is associated with a 4.9–12.8% rate of SLN metastases. However, according to these studies, only 0–2.3% of melanomas ˂0.76 mm is associated with nodal disease. Our results are similar for thin melanomas <1 mm (11.5%) but we have found a relatively high positive sentinel rate (8%) for primary melanomas ˂0.8 mm. While Murali et al. [15] reported that patients with thin melanomas of <0.50 mm have negative SLN stage, Bagaria et al. reported that melanomas of <0.50 mm are identified as a factor of worse prognosis in term of SLN metastases [22]. In our series, 1/9 cases of primary melanoma ˂0.50 mm had nodal metastasis. Interestingly, corresponding to results by Mitteldorf et al. [9], we found no significant difference in sentinel nodal metastases between the ˂0.76 mm and the 0.76–1.00 mm groups.

### Ulceration

According to the latest two AJCC Melanoma Staging and Classification schemes, thin melanomas continue to be classified as T1b by the presence of ulceration [4, 13]. Several studies have reported that ulceration is a rare event (1–15%) in thin melanomas [3, 23–25]. In our series, only 12 melanomas were ulcerated among the 152 pT1 tumours (7.9%). In the group of patients that underwent SNB, 8/78 primary tumours (10%) showed ulceration. In their study of 147 thin melanoma patients, Yonick et al. found that ulceration (and Breslow thickness) was an independent predictor of nodal disease [26]. A study of 77 patients by Oliveira Filho et al. confirmed this result [27]. However, most studies have not shown ulceration as a significant predictor [10, 11, 15, 26, 28–30]. Kesmodel et al. reported that 181 thin melanoma patients with positive SLNs showed no signs of ulceration in the primary tumour [28]. Our results are consistent with these findings. None of the primary thin melanomas showing ulceration had nodal metastasis.

### Mitotic Rate

Mitotic rate (MR) is defined as the maximum number of dermal mitoses per mm^2^. According to the staging system for melanomas in the AJCC7^th^ edition, even a single mitosis can be categorized as T1b in the case of a small dermal tumour area, and, therefore, SLB should be considered [4, 31]. However, with only one mitotic figure being the cut-off point, this method may be unreliable even with an additional immunohistochemistry [32]. Several authors have reported that primary melanoma mitoses predict SLN status [10, 16, 28]. Furthermore, Sondak et al. have found young patients (<35 years) that showed an MR correlation with a positive SLN. Interestingly, this was not the case with Breslow thickness [16]. Other studies showed no such association, even revealing that up to one-third of SLN-positive thin melanomas have zero mitosis [3, 15, 33]. In our study, there was no observable significance in mitotic rate between the node-positive and -negative group (Student t-test; t = −0.688; df = 76; *p* = 0.494).

### Regression

**Fig. 1:**
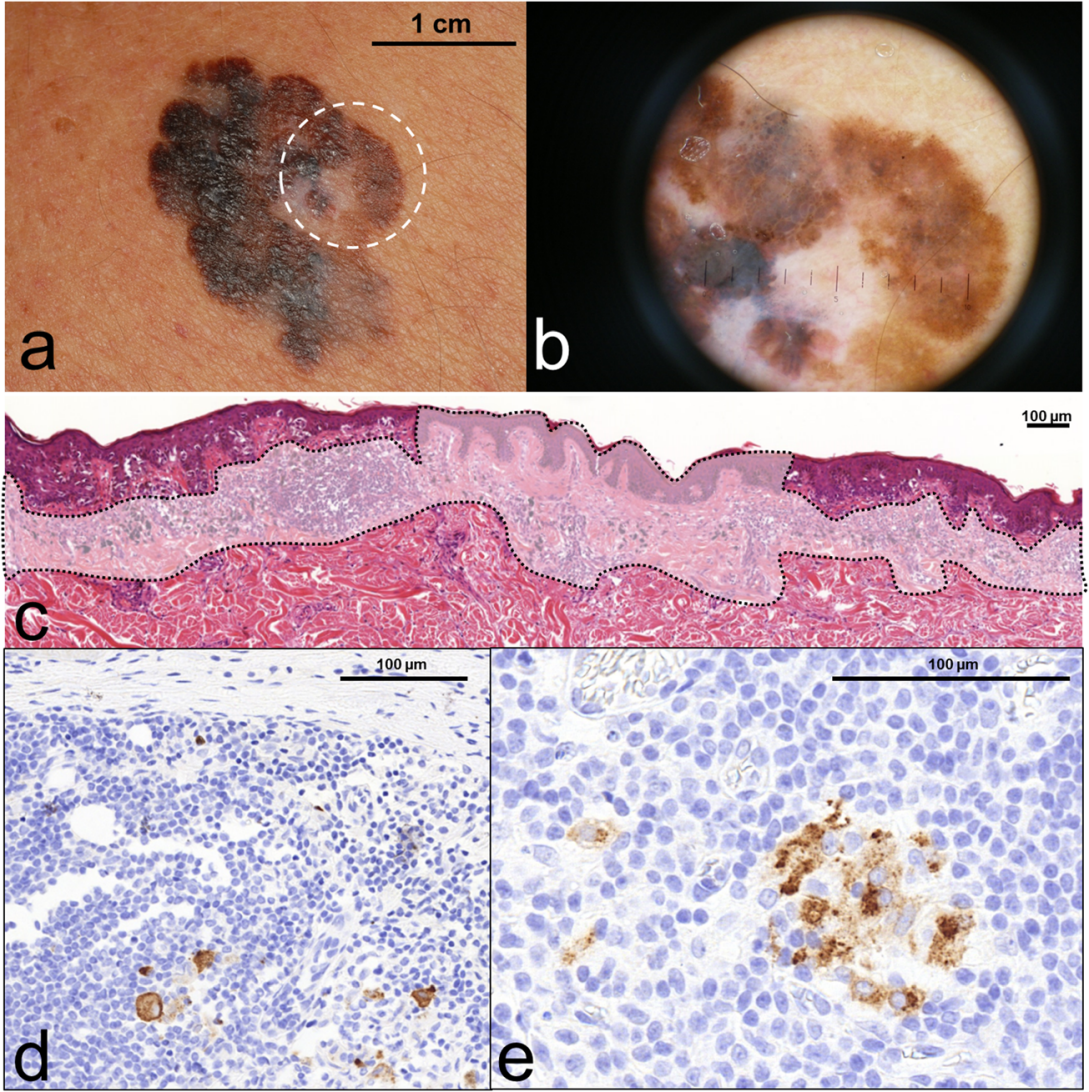
shows a case report of a male patient aged 71, presenting regressing superficial spreading melanoma on his back region (**a**). At dermoscopy, the centre of the polychrome plaque displayed greyish-whitish area with peppering sign which is characteristic for regression (**b**). After the surgical removal of tumour, the histopathology showed extensive vanishing of junctional and dermal melanoma cells replaced by fibrosis, accumulation of melanophages and lymphocytic infiltrate together with focal neovascularisation (**c** - circumscribed faded area). Only the edges of the presented section contained atypical residual microinvasive melanoma cells within the regressive microenvironment. Although calculated Breslow thickness from the residual melanoma counterpart showed only 0.532 mm, dermal mitotic activity together with the adverse regression indicated SLNB. During the histopathological processing right axillary SLN contained scattered metastatic melanoma cells (**d**) which were also present in two other lymph nodes in the right axillary dissection sample (**e**)

The clinical significance of clinical and histological regression in melanoma is still debated, with numerous studies reporting a higher rate of metastasis in thin, regressed melanomas [34, 35]. In our previous study [30], we also found that tumour regression predicts a higher risk of sentinel node involvement in melanomas ˂2.0 mm in a series of 134 melanoma patients. Other authors showed no association with recurrence or survival [18, 36]. This contradiction could be explained in part with the lack of a uniform definition for regression. Without these uniform criteria, the reproducibility of the results may be difficult. At our department, we used the criteria suggested by the Pathological Group of the World Health Organization Melanoma Programme for the definition of regression. This includes the presence of a zone of tumour-free epidermis and dermis in which there is fibrosis, often along with inflammation and dilated vessels, flanked on one or both sides by a tumour (Fig. 1). These criteria for regression are also involved in the study by Botella-Estrada et al. [37] regarding (i) decrease or absence of melanoma cells in the dermal component of the tumour, presence of (ii) fibrosis, (iii) inflammatory infiltrate, (iv) melanophages, (v) neovascularization, (vi) epidermal flattening and (vii) keratinocytic/melanocytic damage. Features (i–v) together are considered obligatory elements for the diagnosis of regression. The extension of regression was horizontally evaluated and divided into focal or main categories; the cut-off point was a percentage of 75% in the horizontal extension of primary melanoma.

In our recent study using univariate and multivariate logistic regression analysis, regression turned out to be the only significant independent predictor of SLN metastases (OR = 5.796). These results confirm our previous findings that patients with Breslow <2.0 mm but regressing melanomas have a four-time higher relative risk of developing nodal metastases than patients with non-regressing melanomas. This may verify our previous hypothesis that histological regression can result in a decreased Breslow thickness measurement and thus in some cases an erroneously more favourable prognostic estimate. In contrast, the overvaluation of early regression signs may also result in false regression data for statistical analyses.

## Conclusion

According to the large, multicentre studies, thin melanomas (<1.00 mm) have a low, but significant risk for SLN metastases; however, these studies often apply various criteria for staging and performing SNB. Prospective standardized multicentre trials with standardized clinicopathologic and demographic criteria for performing SNB in thin melanomas are needed to specify widely accepted, reliable predictors.

Compared to the numerous multicentre-studies published recently, the benefit of our results may be the uniform criteria for diagnosis and treatment, as all the cases were managed at a single institution, with uniform surgical techniques and standard histology protocols for processing and evaluating primary melanomas and SLNs.

Our analysis support the recent AJCC8^th^ classification that mitotic rate alone is not a sufficiently powerful predictor of SLN status in thin melanomas. If strict histopathological definition criteria are applied, regression might be an additional adverse feature that aids to identifying those T1 patients most likely to be SLN-positive, therefore sentinel lymph node biopsy might be considered in the case of patients with widely regressive thin (˂0.8 mm) melanomas.
